# Norton’s Ophthalmic Therapeutics

**Published:** 1882-07

**Authors:** George H. Clark

**Affiliations:** West Walnut Lane and Green Street, Germantown, Phila.


### NORTON'S OPHTHALMIC THERAPEUTICS.

*To the Editor of* THE HOMŒOPATHIC PHYSICIAN:

In your journal for June appears a criticism of my review of Dr. Norton's recent work on "Ophthalmic Therapeutics," signed Charles Deady, M. D. In reply, I would simply say that Dr. Deady's letter does not in any way consider the point at issue. In my review of the work I wished simply to point out the excellencies of Part I, and the eclecticism of Part II. Dr. Deady, in his attempted defense of Part II, says:

"I wish to have it distinctly understood in starting that I am no lover of external applications, but a firm believer in the efficacy of the indicated remedy, and perfectly willing to give it in a high potency and follow it with Sac. lac., if such shall appear to be the best treatment for the case. From this standpoint I do not feel disposed to take issue with G. H. C. in his condemnation of local applications in general, but would only remind him that in a large dispensary practice we occasionally meet with cases with so little vitality that they fail to react to even the best selection of remedies in any and all potencies, and that sometimes in such cases an external application seems to start a reaction which we can afterwards follow up with internal medication."

As the rest of Dr. Deady's communication consists simply of figures showing how the allopaths maltreat their cases, I shall omit it in my discussion of a homœopathic book; satisfying myself with a few comments upon the sentences quoted. Before making any comments I must express the great pleasure I feel in learning that Dr. Deady is "a firm believer in the efficacy of the indicated remedy," and I am glad that he distinctly stated this fact, as the treatment he advises gives little evidence of it.

1. Dr. Deady professes to condemn local applications save in large dispensary practice. In my review I presumed that Norton's work was written for private as well as dispensary practice; but if the author intended the homœopathic portion for private, and the eclectic for dispensary practice, he should have so stated. Even in dispensary practice, Dr. Deady admits that cases needing (eclectic) local applications are only "occasionally met" and that "in such cases" an external application only "*sometimes*" "*seems*" to start a reaction. This, then, is the best apology he can make for such treatment. This work, written for the daily guidance of the private practitioner, recommends as the best treatment, measures which the apologist admits are only "*occasionally*" needed in a large dispensary practice, and even then "*seem*" only "*sometimes*" to be beneficial.

2. It is entirely unnecessary for me to attempt to prove to a homœopathist that cases do not occur, even "in a large dispensary practice," where the vitality is so low that the patient fails "to react to even the best selection of remedies," in a curable case; if any refutation were necessary mere mention of the reaction in cases of cholera or typhoid fever would be sufficient. As to "a large dispensary practice" making eclecticism necessary, I have nothing to say, the excuse is absurd. In conclusion, I may as well mention that the several cases of gonorrhœal ophthalmia, named in my review, occurred during the course of several years, in the clinics of the Hahnemann Medical College, of this city, when the chair of Clinical Surgery was so ably filled by Prof. Macfarlan, and during which I was his assistant, in charge of the ophthalmic patients.

Indeed, it appears to me that Dr. Deady was scarcely wise in attempting any defense of such practice; for he merely calls attention to objectionable features which might never have been noticed.

G. H. C. wishes to assure Dr. Deady of his most distinguished consideration, and he would be pleased to have him and his *chef* again put their heads together—two heads *are* better than one—and inform him of whom the allopaths thus write: "If, for the purpose of securing patronage, the homœopath pretends to a superior system, in which he does not believe, and to a better practice which he does not follow, he is a charlatan and a pretender, unworthy of confidence and honorable associations." (*North American Review, March*, 1882.)

George H. Clark,
*West Walnut Lane and Green Street, Germantown, Phila.*

